# Liver function assessment using indocyanine green plasma disappearance rate in a young male with icteric leptospirosis: a case report

**DOI:** 10.1186/s12879-019-4101-5

**Published:** 2019-05-28

**Authors:** Alicja J. Kunikowska, Monika Wildgruber, Ewert Schulte-Frohlinde, Tobias Lahmer, Roland M. Schmid, Wolfgang Huber

**Affiliations:** 1 Klinik und Poliklinik für Innere Medizin II, Klinikum rechts der Isar, Technische Universität München, Ismaninger Straße 22, D-81675 Munich, Germany; 2 Innere Medizin I, Klinikum Freising, Alois-Steinecker-Straße 18, D-85354 Freising, Germany

**Keywords:** Liver failure, Leptospirosis, Liver function tests, Indocyanine green, Plasma disappearance rate, Bilirubin

## Abstract

**Background:**

Leptospirosis is one of the leading global zoonotic causes of morbidity and mortality. It is induced by a pathogenic spirochete of the genus Leptospira. The icteric form of leptospirosis is characterized by pronounced hyperbilirubinemia and associated with significantly increased mortality. Conventional static liver function tests insufficiently assess hepatic damage and have limited prognostic value. Dynamic tests, such as indocyanine green plasma (ICG) clearance, more adequately reflect hepatic functional status. In this case report we describe the ICG plasma disappearance rates (ICG-PDR) in a patient with leptospirosis and massive hyperbilirubinemia, expanding our knowledge of liver dysfunction in icteric leptospirosis.

**Case presentation:**

A 21-year-old Caucasian man presented with acute-onset jaundice, myalgia, fever and headaches. Laboratory tests upon admission revealed, most notably, acute kidney failure and hyperbilirubinemia of 17 mg/dl with mild elevation of aminotransferases. In the course of the following 4 days, total serum bilirubin increased to 54 mg/dl. The clinical outcome was favorable with intravenous ceftriaxone and doxycycline. Presumptive diagnosis of leptospirosis was later confirmed by PCR-based amplification of leptospiral DNA in the blood. ICG-PDR values, bilirubin as well as aminotransferases were recorded throughout hospitalization and a 3-month follow-up period. Initially dramatically reduced ICG-PDR (2.0%/min, normal range: 18–25%/min) rapidly normalized within 10 days, while bilirubin remained elevated up to week 7. Mild elevation of serum alanine aminotransferase was at its peak of 124 U/l by day 12 and reached close to normal levels by week 7 upon admission.

**Conclusions:**

Markedly diminished ICG-PDR values presented in this case report suggest severe liver function impairment in the acute phase of icteric leptospirosis. Prolonged elevation of serum bilirubin may not adequately reflect recovery of liver injury in this disease. ICG clearance appears to be a promising marker for the detection of hepatic dysfunction and recovery in icteric leptospirosis in addition to the static tests.

**Electronic supplementary material:**

The online version of this article (10.1186/s12879-019-4101-5) contains supplementary material, which is available to authorized users.

## Background

Leptospirosis is a global and highly prevalent zoonotic disease of substantial and still increasing epidemiological importance, especially in the developing countries [1–3]. As of 2015, global incidence of leptospirosis amounted to 1.03 million cases annually with 58,900 deaths worldwide [3]. It is caused by a spirochete of the genus Leptospira [2]. The most important reservoirs of pathogenic leptospires are small mammals which intermittently shed the bacteria with urine [2]. From so contaminated water and soils leptospires can enter the bloodstream of humans, their accidental hosts, via discontinued skin or mucosa [1, 2].

Clinical manifestation of leptospirosis ranges from mild febrile syndrome to multi-organ failure [2]. Following incubation period of 7–12 days, leptospirosis usually presents itself with sudden onset of fever, myalgia, and headache while other common symptoms include unproductive cough, nausea, vomiting, diarrhea, and abdominal pain [2]. Jaundice, hemorrhagic diathesis, signs of renal or pulmonary failure, and altered mental status may indicate failure of various organ systems [2]. In a mild form of leptospirosis, routine laboratory blood test results are usually non-specific [4]. In severe leptospirosis, as a result of interstitial nephritis, elevated plasma creatinine and unique potassium wasting high-output renal dysfunction due to inhibition of Na^+^-K^+^-Cl^−^ co-transporter in the Henle loop, are commonly observed [4, 5]. In icteric leptospirosis, characterized by rapidly progressive clinical course, highly elevated serum bilirubin accompanied by moderate increase in hepatic transaminases may occur [2]. The combination of jaundice and renal failure is commonly referred to as Weil’s disease [4].

Of all leptospiral cases, 5–10% develop the severe, icteric form [4] which is associated with mortality rate of 5 to 15% [6]. It remains unclear to what degree the overall liver function is diminished in icteric leptospirosis [6]. Bilirubin and other commonly used parameters of hepatic function such as aminotransferases, albumin or prothrombin time, are static estimates unable to assess complex liver functions such as clearance of substances or formation of metabolites [7]. Recently, dynamic tests quantifying the ability of the liver to eliminate or metabolize certain substances and thus reflecting its functional status, have been subject of increasing interest due to their prognostic potential in early detection of liver failure [7, 8].

Indocyanine green (ICG) clearance assessment is the most commonly used dynamic liver function test performed at bedside [9]. After intravenous injection, ICG is selectively taken up by hepatocytes in an ATP-independent process [9, 10]. ICG does not undergo intracellular metabolization and its excretion into the bile occurs ATP-dependently, making its clearance a suitable proxy for hepatocellular energy status [10]. Since the molecule does not participate in the enterohepatic circulation, its elimination kinetics — usually expressed as plasma disappearance rate — depends on liver blood flow, parenchymal cellular function, and biliary excretion [7, 9, 10]. The test is performed with the use of transcutaneous pulse-densitometry, providing results within 6–8 min [7, 10].

It has been reported that global incidence of leptospirosis is on the rise due to expansion of urban slums as well as climate changes [3]. The alarming data on morbidity and mortality prompt us to further investigate the pathogenesis of this disease and to identify its prognostic markers. The so far identified and validated poor prognostic factors for leptospirosis include age (> 40 years), oliguria, respiratory insufficiency, pulmonary hemorrhage, cardiac arrhythmias, and altered mental status [2, 4]. Although the degree of jaundice has not been proven to have prognostic importance, the mere presence of jaundice appears to define the course of disease, since virtually all leptospirosis deaths occur in icteric patients [6].

Our report aims to advance our understanding of liver dysfunction in icteric leptospirosis using the state-of-the-art method of assessing hepatic substance clearance. We performed a series of measurements of ICG plasma disappearance rate (ICG-PDR) in a young male suffering from icteric leptospirosis during initial hospitalization and a 3-month follow-up period. To the best of our knowledge, this is the first published measurement of ICG-PDR in documented leptospirosis.

## Case presentation

A 21-year-old Caucasian male was admitted to a hospital (collaborating institution) in Southern Germany in late summer with newly manifested jaundice as well as a seven-day-history of myalgia, retro-orbital headaches, fatigue, recurrent fever, and nausea. Since the beginning of his illness, myalgia — especially in the calf region — intensified, causing the patient an increasing difficulty in walking. He reported an episode of gum bleeding after cleaning his teeth. There was no history of traveling abroad in the last months and no recent contact with animals. Two weeks prior to the onset of his symptoms the patient sustained a minor knee injury resulting in a skin abrasion while bathing in the river Isar close to Munich, Germany.

The patient works as a computer scientist and has no relevant medical history. Weight and height upon admission were recorded to be 90 kg and 189 cm, respectively (BMI = 25.2 kg/m^2^). Alcohol, nicotine, or drug anamnesis was negative. No medication or allergies were reported.

On examination, the patient appeared tired, but he displayed no neurological abnormalities. Body temperature was 36.8 °C, pulse 90 bpm, blood pressure 114/75 mmHg, respiratory rate 16 bpm, and oxygen saturation 99% while breathing ambient air. The lungs and heart auscultation was unremarkable, the abdomen was soft and non-tender. The skin and scleral inspection revealed jaundice and a slight gum bleeding was observed during the examination of the oral cavity. Upon pressure, tenderness in the thighs and calves was reported.

Abdominal ultrasound upon admission to the hospital revealed hepatosplenomegaly and no signs of intra- or extrahepatic cholestasis. White-cell count was 9.9 G/L (87% neutrophils and 3.5% lymphocytes), platelet count 39 G/L, and hemoglobin 13.3 g/dl. Serum sodium level was 123 mmol/l, potassium 3.15 mmol/l, and creatinine 2.8 mg/dl (248 μmol/l) with glomerular filtration rate of 31 ml/min/1.73m^2^. C-reactive protein was 15.4 mg/dl, procalcitonin 2.86 ng/ml, and interleukin-6 was 66.4 pg/ml. Aspartate aminotransferase was 92 U/L, alanine aminotransferase 65 U/l, total bilirubin 17.3 mg/dl (with direct bilirubin reaching 14.7 mg/dl), gamma-glutamyltransferase 47 U/l and alkaline phosphatase 108 U/l. Creatine kinase was 1197 U/l, prothrombin time international normalized ratio 1.2, and partial-thromboplastin time 33 s. Urinalysis revealed a pH of 8, 3+ protein (6.3 g protein/12 h), 5–10 leukocytes per high power field, and 0–2 erythrocytes per high power field. An overview of laboratory test results with the corresponding reference ranges is shown in Table 1.

**Table 1:** Laboratory data during hospitalization. Day 1–4: other hospital, Day 5–12: this hospital

Variable	Reference Range^a^	Day 1^a^	Day 2^a^	Day 4^a^	Reference Range^b^	Day 5^b^	Day 8^b^	Day 12^b^
Hematocrit [%]	*39.5–50.5*	37.4	35.9	30.2	*40–48*	26.4	26.4	26.3
Hemoglobin [g/dl]	*13.5–17.2*	13.3	13.2	11.0	*14–18*	9.7	9.2	8.5
White-cell count [G/l]	*3.9–10.2*	9.9	9.9	18.7	*4–9*	17.4	16.78	6.56
Platelet count [G/l]	*150–370*	39	42	101	*150–450*	86	66	240
C-reactive protein [mg/dl]	*< 0.5*	15.4	9.4	*–*	*< 0.5*	3.6	0.5	–
Creatinine [mg/dl]	*0.7–1.2*	2.80	4.29	1.67	*0.7–1.3*	1.2	0.9	0.8
Estimated GFR^c^ [ml/min/1.73^2^]	*> 60*	31	18	58	*> 60*	> 60	> 60	> 60
Sodium [mmol/l]	*135–146*	123	117	133	*135–145*	137	134	139
Potassium [mmol/l]	*3.5–5.1*	3.15	2.60	4.47	*3.5–5.0*	4.2	4.1	4.1
Calcium [mmol/l]	*2.00–2.65*	2.17	2.02	2.08	*2.20–2.65*	2.06	*–*	–
Creatine kinase [U/l]	*< 190*	1197	881	137	*< 174*	87	*–*	–
Aspartate aminotransferase [U/l]	*< 50*	92	73	55	*10–50*	69	58	76
Alanine aminotransferase [U/l]	*< 50*	65	66	51	*10–50*	53	83	124
Gamma-glutamyltransferase [U/l]	*< 60*	47	37	31	*< 66*	29	22	18
Alkaline phosphatase [U/l]	*40–130*	108	104	95	*40–129*	87	*–*	–
Total bilirubin [mg/dl]	*0.1–1.2*	17.27	27.04	53.88	*< 1.2*	44.4	15.1	6.2
Direct bilirubin [mg/dl]	*< 0.2*	14.72	23.09	38.94	*< 0.3*	44.4	*–*	–
Albumin [g/dl]	*3.5–5.2*	3.6	2.7	3.0	*3.5–5.0*	2.8	*–*	*–*
Quick value [%]	*70–130*	76	71	98	*70–120*	103	*–*	–
Prothrombin time: INR^d^	*0.95–1.15*	1.2	1.1	1.0	*–*	1.0	*–*	–
Partial thromboplastin time [sec]	*20–40*	33.4	31.9	36.1	*26–37*	35	*–*	–

^a^Laboratory reference ranges for individual blood test variables obtained in the other hospital

^b^Laboratory reference ranges for individual blood test variables obtained in this hospital

^c^glomerular filtration rate

^d^international normalized ratio

On day 1 upon admission an empiric intravenous antimicrobial regime with doxycycline (100 mg every 24 h) and ceftriaxone (2 g every 24 h) was initiated while a selection of tests in search for microbial and viral agents was pending. The patient received intravenous fluid and electrolyte replacement. Within 48 h upon admission, hypokalemia (2.32 mmol/l) and hyponatremia (113 mmol/l) worsened significantly without signs of acid-base dysregulation in blood gas analysis. Creatinine increased to 4.37 mg/dl (386 μmol/l), and a polyuria with up to 7.2 L urine output /24 h was documented. The patient was transferred to the intensive care unit (ICU) at the initial collaborating institution and — approximately 48 h later — transferred to the ICU at our university hospital. Starting from day 4 since the initial admission creatinine levels started to decrease, polyuria was in regress and potassium and sodium levels normalized. In addition, platelet count improved and C-reactive protein decreased. The patient remained afebrile. Despite the gradually improving general condition of the patient, serum bilirubin levels were constantly rising starting from day 1 since admission, reaching its maximum on day 4 (54 mg/dl total bilirubin, 39 mg/dl direct bilirubin). Aspartate and alanine aminotransferases, alkaline phosphatase and gamma-glutamyltransferase remained normal or mildly elevated (Table 1).

Urine and blood cultures revealed no growth. Screening tests for hepatotropic viruses (hepatitis A, B, C, and E) as well as HIV, Epstein-Barr virus, and cytomegalovirus were negative. Elevated IgG antibodies to herpes simplex virus (HSV 1 and 2) were detected while IgM antibodies remained within the normal range, suggesting an earlier or latent infection with HSV virus. Quantitative serology assays for hantavirus, Chlamydia, Brucella, and Rickettsia (*rickettsii/conorii*) showed no evidence for infection with these pathogens. Urine test for Legionella antigen was negative. Within normal range were: antinuclear antibodies (ANA), antimitochondrial antibodies (AMA), anti-liver/kidney microsomal antibodies type 1 (anti-LKM-1), anti-soluble liver antigen antibody/liver-pancreas (a-SLA/LP), anti-liver membrane antibodies (LMA), anti-liver specific protein antibodies (LSP), and perinuclear- and cytoplasmic-antineutrophil cytoplasmic antibodies (p-ANCA and c-ANCA) whereby autoimmune hepatitis could be ruled out. Normal levels of serum copper (110 μg/dl, reference range: 70–140 μg/dl) as well as serum ceruloplasmin (0.44 g/l, reference range: 0.2–0.6 g/l) allowed to dismiss Wilson’s disease from diagnostic consideration.

The presumptive diagnosis of icteric leptospirosis was strengthened by detection of antileptospiral IgM antibodies with the use of ELISA in the acute phase serum specimen (38 U/ml, reference range: < 15 U/ml, SERION ELISA classic Leptospira IgG/IgM, Virion Serion, Würzburg, Germany) and confirmed by an accredited PCR method using proprietary primers at the Institute for Medical Microbiology and Hygiene at the University of Regensburg, Germany. The therapy with doxycycline was discontinued (total duration of 3 days) and the regime with ceftriaxone carried on as a targeted monotherapy (total duration of 12 days).

The patient was discharged home after a total of 12 days of hospitalization (3 days at the ICU). The general condition upon discharge was reported as good; fatigue was declining and the remaining symptoms, with the exception of jaundice (total bilirubin 6.2 mg/dl), subsided.

**Fig. 1:**
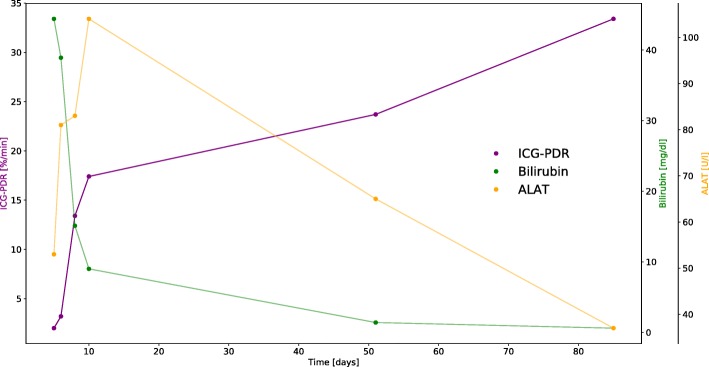
Indocyanine green plasma disappearance rates (ICG-PDR) and serum total bilirubin as well as alanine aminotransferase (ALAT) levels during hospitalization and a 3-month follow-up period

Throughout hospitalization and a 3-month follow-up period, indocyanine green plasma disappearance rates were recorded multiple times. For each measurement, 25 mg of the ICG dye were dissolved in 5 ml of distilled water, and a dose of 22.5 mg (0.25 mg/kg body weight) was injected intravenously. ICG-PDR was determined via non-invasive, transcutaneous pulse dye densitometry with the use of the LiMON device (Pulsion Medical Systems SE, Feldkirchen, Germany). Initial concentration at time “0” was set to be 100% and plasma disappearance rate was calculated as percentage change over time (%/min) [7]. Normal values for ICG-PDR are considered to be 18–25%/min [7, 10]*.* The measured ICG-PDR values, total bilirubin and alanine aminotransferase serum levels are displayed in Fig. 1.

Initially severely reduced ICG-PDR (2%/min on day 5 upon initial admission) gradually improved within several days to reach almost normal level on day 10 upon initial admission to hospital (17.4%/min). Approximately 7 weeks after initial hospitalization, ICG-PDR was recorded to be within normal range (23.7%/min) and reached 33.4%/min on day 85. While ICG-PDR values rapidly normalized in parallel with clinical improvement, serum bilirubin levels were slowly decreasing (44.4 mg/dl on day 5 and 9.0 mg/dl on day 10 upon hospitalization), and it was only until 7 weeks upon admission that they reached normal levels (Fig. 1). Mild elevation of serum alanine aminotransferase was at its peak of 124 U/l on day 12 upon hospitalization (Table 1) and reached close to normal levels by week 7 upon admission (Fig. 1 and Additional file 1). Within the 3-month follow-up period, fatigue resolved and the patient reported no remaining symptoms.

## Discussion and conclusions

Our case report documents the indocyanine green plasma disappearance rates in an individual with icteric leptospirosis during the initial hospitalization phase and a follow-up period of 3 months. We observed critically low ICG-PDR values during acute hyperbilirubinemic phase of infection with only mild elevation of hepatic transaminases, normal prothrombin time and mildly reduced albumin levels (Table 1, Fig. 1). These results demonstrate a substantial reduction in liver function in icteric leptospirosis despite the almost unremarkable remaining standard laboratory tests. In our report, rapidly improving levels of ICG-PDR were accompanied by slowly subsiding bilirubinemia, demonstrating that prolonged elevation of serum bilirubin may not adequately reflect liver dysfunction in Weil’s disease. With the increasing accessibility of ICG-PDR measurement and its easy-to-use, bedside- and non-invasive character, it is a promising parameter of liver dysfunction in individuals suffering from icteric leptospirosis. In regions where this disease is endemic and intensive care is scarce, a fast and reliable assessment whether ICU admission is indicated, may be of great value.

So far, multiple studies have demonstrated that ICG clearance measurement allows an early and sensitive detection of liver dysfunction, proving to be a superior prognostic marker to static tests such as serum bilirubin or hepatic transaminases [7]. ICG elimination was shown to be a more reliable marker in graft function prediction of liver transplants than the use of serum enzyme markers [11]. Also, ability to eliminate ICG has been demonstrated to constitute an early indicator of reversible liver injury in septic shock [8]. Furthermore, ICG-PDR proved to be a good predictor of survival in critically ill patients: sensitivity as well as specificity of ICG-PDR upon admission were comparable to those of well-established and complex scores such as APACHE II and SAPS II [7]. A need for studies investigating whether ICG-PDR is a suitable prognostic marker in icteric leptospirosis still exists.

In conclusion, in this unprecedented case report, we demonstrate severe liver function impairment in the acute phase of icteric leptospirosis measured with indocyanine green plasma disappearance rate. ICG-PDR rapidly improved despite prolonged elevation of serum bilirubin levels, suggesting that serum bilirubin may not adequately reflect hepatic recovery in icteric leptospirosis. Further investigation of prognostic potential of ICG clearance in this highly prevalent disease is needed.

## Additional file

Additional file 1: Numeric values of indocyanine green plasma disappearance rates (ICG-PDR) and serum total bilirubin as well as alanine aminotransferase (ALAT) levels during hospitalization and a 3-month follow-up period. (XLSX 9 kb)

## Abbreviations

ALAT: Alanine aminotransferase
ICG-PDR: Indocyanine green plasma disappearance rate
ICU: Intensive care unit
